# A novel subtype based on driver methylation–transcription in lung adenocarcinoma

**DOI:** 10.1007/s00432-024-05786-3

**Published:** 2024-05-22

**Authors:** Xin Wang, Zhenyi Xu, Shuang Zhao, Jiali Song, Yipei Yu, Han Yang, Yan Hou

**Affiliations:** 1 Clinical Trial Research Center, Beijing Hospital, National Center of Gerontology, Institute of Geriatric Medicine, Chinese Academy of Medical Sciences, Beijing, 100730 China; 2 Huadong Hospital Affiliated to Fudan University, Shanghai, 200040 China; 3 Department of Biostatistics, School of Public Health, Peking University, Beijing, 100191 China; 4 Key Laboratory of Carcinogenesis and Translational Research (Ministry of Education), Department of Lymphoma, Peking University Cancer Hospital & Institute, Beijing, 100142 China; 5 Peking University Clinical Research Center, Peking University, Beijing, China

**Keywords:** Driver methylation gene, Heterogeneity, Prognosis, Personalized therapy, Tumor microenvironment, Lung adenocarcinoma

## Abstract

**Aims:**

To identify driver methylation genes and a novel subtype of lung adenocarcinoma (LUAD) by multi-omics and elucidate its molecular features and clinical significance.

**Methods:**

We collected LUAD patients from public databases, and identified driver methylation genes (DMGs) by MethSig and MethylMix algrothms. And novel driver methylation multi-omics subtypes were identified by similarity network fusion (SNF). Furthermore, the prognosis, tumor microenvironment (TME), molecular features and therapy efficiency among subtypes were comprehensively evaluated.

**Results:**

147 overlapped driver methylation were identified and validated. By integrating the mRNA expression and methylation of DMGs using SNF, four distinct patterns, termed as S1-S4, were characterized by differences in prognosis, biological features, and TME. The S2 subtype showed unfavorable prognosis. By comparing the characteristics of the DMGs subtypes with the traditional subtypes, S3 was concentrated in proximal-inflammatory (PI) subtype, and S4 was consisted of terminal respiratory unit (TRU) subtype and PI subtype. By analyzing TME and epithelial mesenchymal transition (EMT) features, increased immune infiltration and higher expression of immune checkpoint genes were found in S3 and S4. While S4 showed higher EMT score and expression of EMT associated genes, indicating S4 may not be as immunosensitive as the S3. Additionally, S3 had lower TIDE and higher IPS score, indicating its increased sensitivity to immunotherapy.

**Conclusion:**

The driver methylation-related subtypes of LUAD demonstrate prognostic predictive ability that could help inform treatment response and provide complementary information to the existing subtypes.

**Supplementary Information:**

The online version contains supplementary material available at 10.1007/s00432-024-05786-3.

## Introduction

Lung cancer is the leading cause of cancer incidence and mortality in the world, with an estimated 2.2 million new cancer cases and 1.8 million deaths in 2020(Sung et al. 2020). Non-small cell lung cancer (NSCLC) is the most common histologic type of lung cancer, accounting for approximately 80–85%, and with an unfavorable prognosis with a 5-year survival rate of all stages approximately 15% (Chen et al. 2014; Herbst et al. 2018; Jemal et al. 2011).The main histological classification of NSCLC is lung adenocarcinoma (LUAD). Targeted drugs and immunotherapy are promising treatments for LUAD, but only a small proportion of patients benefit from them (Li et al. 2013; Shaw et al. 2013; Zhou et al. 2021; Park et al. 2021; Skoulidis et al. 2021; Lantuejoul et al. 2020; Hirsch et al. 2017; Yarchoan et al. 2017). Further, LUAD patients with similar clinical characteristics may experience different clinical outcomes due to the different molecular features (Niemira et al. 2019). Therefore, further research on LUAD is warranted to identify new therapeutic targets and sensitive subtype to improve prognosis.

DNA methylation is a major epigenetic modification and plays an important roles in carcinogenesis and progression (Fu et al. 2022); and recent evidence has showed that driver methylation rather than passenger methylation are associated with cancer progression and prognosis (Pfeifer 2018; Kalari and Pfeifer 2010). Previous studies have suggested that LUAD could be stratified into different molecular subtypes by methylation signatures (Guidry et al. 2022; Yu et al. 2023; Smet 2023). However, there is lack of evidence on studying the driver methylation-based subtype of LUAD. Further, multi-omics integration is a rapidly evolving area of research that could be of great benefit in better understanding complex diseases (Kreitmaier et al. 2023). Therefore, integrating methylation and mRNA of driver methylation may lead to a comprehensive understanding of LUAD.

This study aims to identify a novel molecular subtype of LUAD based on driver methylation. We collected multiple LUAD cohorts from TCGA and CPTAC database, and systematically analyzed clinicopathologic, biological characteristics and outcomes. The heterogeneity was analyzed to provide a new perspective for improving prognosis and treatment response in LUAD.

## Method

### Public cohorts and data pre-processing

We collected publicly available datasets of multi-omics in lung adenocarcinoma (LUAD) from The Cancer Genome Atlas Project (TCGA) and Clinical Proteomic Tumor Analysis Consortium (CPTAC) (Gillette et al. 2020). In TCGA, we included tumor and normal patients with transcriptomics, epigenetics, genomics and clinical data. The RNA-seq data (Illumina TruSeq) were transformed by log2 (FPKM + 1) and methylation were Illumina Infinium 450 K methylation array, average promoter methylation was defined as − 1,500 bp to + 500 bp windows relative to the transcription start site. In CPTAC, we included tumor patients with transcriptomics, epigenetics and clinical data. The RNA-seq data (Illumina TruSeq) were transformed by log2(RPKM) and methylation were Illumina Infinium 850 K methylation array. Gene expression was illustrated by plotting the median expression value when the genes were with one more probe. Genes with more than 50% equal to 0 value were deleted, and KNN imputation was imputed the remaining missing values.

### Identification and validation of driver methylation genes (DMGs)

In our analysis, we aimed to find the DMGs of LUAD and used MethSig and MethylMix methods (Pan et al. 2021; Cedoz et al. 2018). In MethSig, a beta regression model with relevant covariates was used to estimate expected tumor promoter hypermethylation, and genes whose promoter hypermethylation significantly exceeds expectation regarded as DMGs. MethylMix is constructed by a beta mixture model to identify methylation states and compare with the normal DNA methylation state, finding methylation states with differential and predictive. In TCGA cohort, methylation array was obtained for tumor (n = 439) and normal (n = 32) samples, and mRNA in the normal sample (n = 58) was used to identify the DMGs. The overlapped genes of MethSig and MethylMix were recognized as DMGs of LUAD. LUAD patients in CPTAC cohort was used to validate DMGs.

### Multi-omics integration analysis

The DMGs play an important role in the change of gene expression. Therefore, we integrated methylation and mRNA expression of DMGs with similarity network fusion (SNF) to discover a novel subgroup of LUAD and explore a comprehensive molecular profile. SNF was used to construct patient similarity networks for each omics and fuse these into a representative network. Then spectral clustering was used for unsupervised clustering of representative network (Wang et al. 2014). In TCGA, 439 patients with both methylation and mRNA expression of DMGs were included to find DMGs-associated multi-omics subgroups. And CPTAC LUAD cohort (n = 100) was used to validate the subgroup characteristics. In this study, silhouette coefficient was calculated to compare the clustering performance with information of multi-omics and single-omics.

### Evaluation of molecular features and tumor-infiltrating of immune cells

The differentially expressed DMGs among DMGs associated with multi-omics subtypes were screened out using the R package “limma” (Ritchie et al. 2015). Absolute value of log2 fold change (FC) larger than 1.5 and Benjamini–Hochberg (BH) adj *P*-value < 0.05 were considered as significance criteria. To investigate the important functional phenotypes among different subtypes, we performed gene set enrichment analysis (GSEA) (Subramanian et al. 2005). The “h.all.v2023.1.Hs.symbols.gmt” gene set was used as the reference gene sets and obtained from the MSigDB database (https://www.gsea-msigdb.org/gsea/msigdb/). In GSEA, normalized enrichment score (NES) > 1.5 and false discovery rate (FDR) *P* < 0.05 were regarded as significantly enrichment.

The epithelial-to-mesenchymal transition (EMT) is a critical cell biological process that occurs during cancer development. In our study, we assessed EMT gene signature, including 25 epithelial and 52 mesenchymal marker genes (Chen et al. 2021; Mak et al. 2016). The EMT score of each sample was calculated with formula $\sum_i^N {\frac{M^i }{N}} - \sum_j^n {\frac{E^j }{n}}$. Here, $M$ and $E$ represent the expression of mesenchymal and epithelial marker genes, $N$ and $n$ represent the gene number of mesenchymal marker and epithelial marker, respectively. In addition, EMT associated genes were compared among subtypes.

Tumor microenvironment (TME) played an essential role in tumor initiation, disease progression, and therapeutic efficacy (Klemm and Joyce 2015). We described the relative abundance of 29 functional gene expression signatures in LUAD by introducing the single sample gene set enrichment analysis (ssGSEA) algorithm. The signatures were derived from the study of Bagaev.A et al. (Bagaev et al. 2021). The enrichment score was derived from ssGSEA reflects the relative degree of TME in each patient. Estimation of STromal and Immune cells in MAlignant Tumours using Expression data’ (ESTIMATE) (Yoshihara et al. 2013) was applied to quantify the infiltration of stromal and immune components in tumor.

Molecular and immune features, obtained from Thorsson V et al. (Thorsson et al. 2018), were further assessed the difference among subtypes, including proliferation, wound healing, B-cell receptor (BCR) Shannon (evaluating BCR diversity), T-cell receptor (TCR) Shannon (evaluating TCR diversity), cancer testis antigens (CTA) score, intratumor heterogeneity (ITH), loss of heterozygosity (LOH; number of segments with LOH events and fraction of bases with LOH events, respectively), homologous recombination deficiency (HRD) and mutation load (nonsilent mutation).

### The therapy sensitivity analysis

To further analyze therapy sensitivity among subtypes, immunotherapy predictors and targeted therapy mutation genes were included in our study. Immunophenoscore (IPS) was positively correlated with tumor immunogenicity, ranged from 0 to 10. It has been used to predict the response of immune checkpoint inhibitors (Charoentong et al. 2017). IPS could be downloaded from https://tcia.at. Tumor Immune Dysfunction and Exclusion (TIDE, http://tide.dfci.harvard.edu/), a computational method to predict immune checkpoint blockade response, was developed by Jiang et al. (Jiang et al. 2018). TIDE integrates tumor immune evasion mechanism including T cell dysfunction and T cell exclusion, and has shown superior predicted performance for immunotherapy response. Furthermore, the mRNA expression of immune modulators and immune checkpoints was analyzed in different multi-omics subtypes (Tang et al. 2018). Enhanced comprehension of the identification of driver gene alterations and co-occurring alterations has significantly altered the therapeutic prospects and is associated with prognosis (Tan and Tan 2022; Skoulidis and Heymach 2019). In our analysis, the mutation and co-occurring mutation of EGFR, STK11, TP53 and KRAS were compared among subtypes.

### Statistical analysis

In this study, all data processing was done in the R-4.2.2 software. Categorized variables were described by frequency (n) and proportion (%). Comparison among three group comparison was estimated by Kruskal–Wallis test. Principal component analysis (PCA) was performed to visualize the difference of DMGs-related genes between tumor and normal samples. Kaplan–Meier curve was generated and determined the significance of the differences via the log-rank test. Multivariate Cox regression analysis was used to assess the independent prognostic factor. All statistical test was two-side and *P* < 0.05 was considered statistically significant.

## Result

### Driver methylation genes of LUAD

**Fig. 1:**
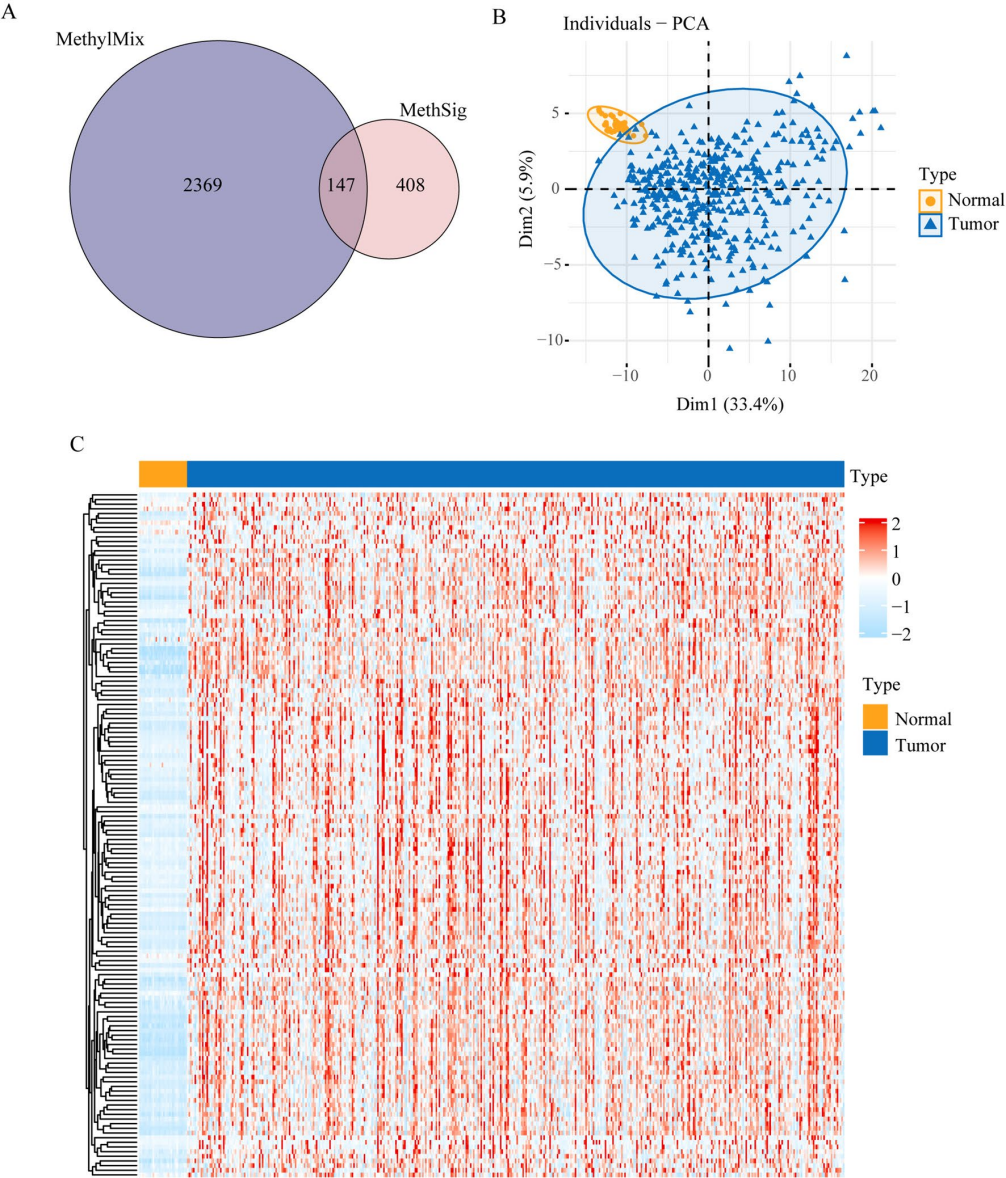
Identification of driver methylation genes (DMGs) in lung adenocarcinoma (LUAD). **A** 147 overlapping DMGs were identified by MethSig and MethylMix in TCGA. **B** Principal component analysis (PCA) reveals a distinct separation between tumors and normal samples based on the expression profiles of DMGs. Tumor samples are represented in blue, and normal samples are represented in yellow. **C** The methylation value of DMGs between normal and tumor tissue in TCGA. The high expression are represented in red, and low expression are represented in blue

In the LUAD cohort of TCGA, 555 driver methylation genes (DMGs) were identified with MethSig and 2,516 DMGs were discovered by MethylMix. A total of 147 overlapped genes were regarded as DMGs of LUAD (Fig. 1A). The principal component analysis (PCA) analysis revealed that the DMGs could effectively distinguish between normal and tumor tissues in TCGA (Fig. 1B). We compared methylation statues of DMGs in tumor and nonmalignant lung tissue and high methylation value were observed in tumor tissue (Fig. 1C). In addition, the correlation between methylation and mRNA expression of DMGs was assessed by Pearson or Spearman correlation analysis (Table S1). Most of DMGs exhibited a negative correlation, and the correlations were statistically significant in TCGA. Furthermore, the DMGs were validated by an independent LUAD cohort from CPTAC, and statistically significant negative correlations between mRNA and methylation were found through Pearson or Spearman correlation analysis (Table S2).

### Prognosis and clinical features of DMGs-associated multi-omics subtypes

**Fig. 2:**
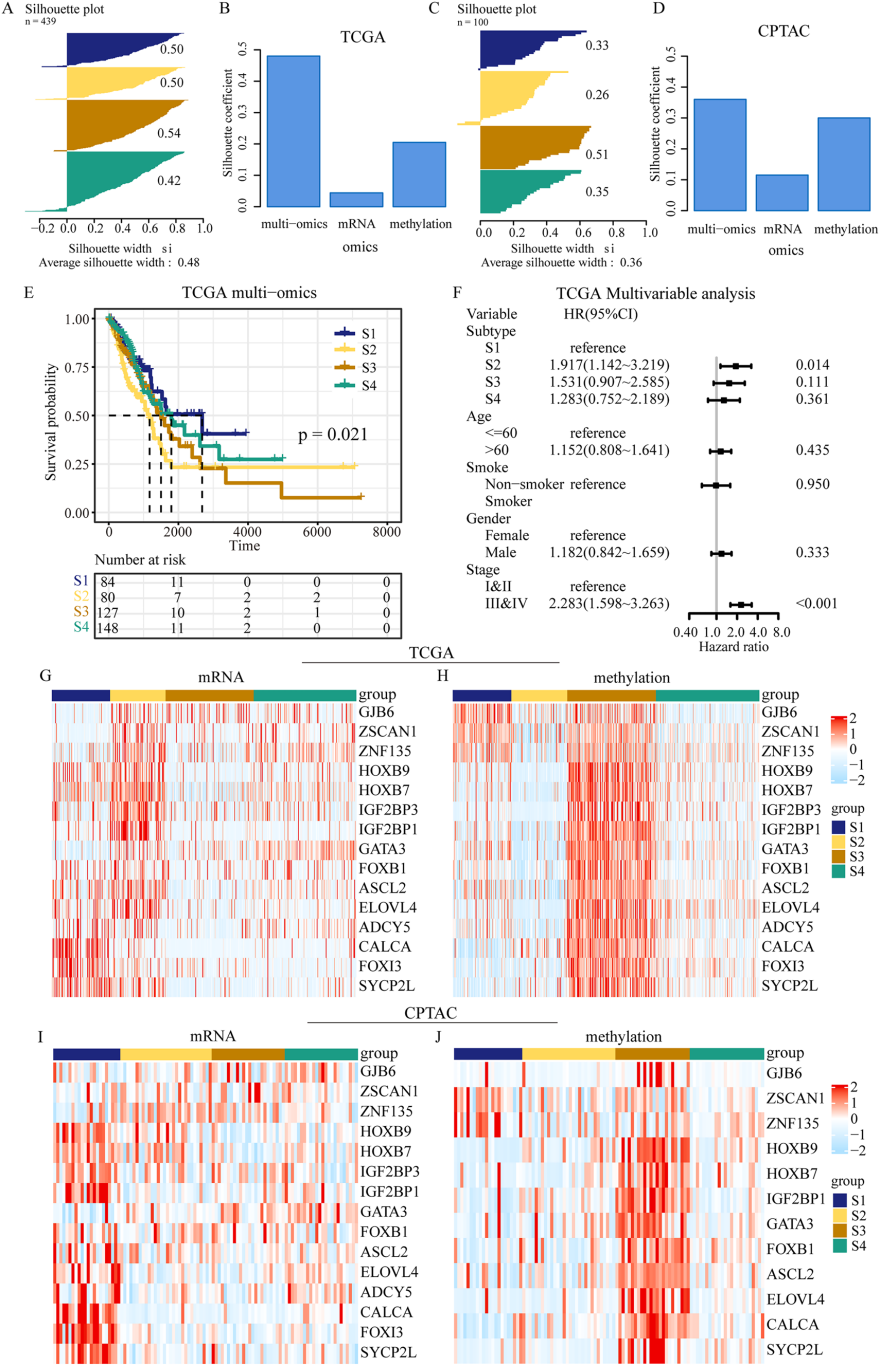
Driver methylation genes (DMGs) associated multi-omics subtypes reveals distinct prognosis and expression patterns in LUAD. **A**, **C** Silhouette coefficient values of samples in multi-omics subtypes in TCGA and CPTAC, respectively. **B**, **D** Silhouette coefficients of the multi-omics unsupervised clustering by similarity network fusion (SNF), and silhouette coefficients of single-omics unsupervised clustering by K-Means in TCGA and CPTAC, respectively. **E** Kaplan–Meier (K–M) curves for overall survival (OS) of different four subtypes (log-rank test, *P* = 0.021). **F** Forest plots illustrate the results of multivariate Cox proportional hazards model of clinical features. **G**–**H** The heatmap shows the mRNA and methylation expression of 15 differential DMGs identified by LIMMA method among the four subtypes in TCGA. **I**–**J** The heatmap shows the mRNA and methylation expression of overlapping differential DMGs among the four subtypes in CPTAC

In our analysis, we integrated methylation and mRNA of DMGs by SNF and the patients were divided into the four molecular subtypes, termed as S1, S2, S3 and S4. In TCGA, we observed that the silhouette coefficient calculated by SNF was higher than that of the K-Means clustering in single-omics data, indicating that multi-omics data exhibited better clustering performance (Fig. 2A–B). In LUAD cohort of CPTAC, the results of silhouette coefficient further confirming that the multi-omics clustering outperformed the single-omics clustering (Fig. 2C–D).

Furthermore, the survival differences among multi-omics subtypes were greater than those among single-omics subtypes (Fig. 2E, Figures S1A–B). We found differences in the prognosis of the molecular subtypes. Patients in S2 tended to have a poorer prognosis in TCGA (*P* = 0.021; Fig. 2E), and the difference between S1 and S2, as well as S2 and S4, were found to be statistically significant with Benjamini–Hochberg method. And the multivariate Cox hazards model also indicated that S2 has worse prognosis tendency than S1 (HR = 1.917, 95%CI: 1.142 ~ 3.219, *P* = 0.014; Fig. 2F). Further comparing the clinical characteristics among subtypes, we found statistically significant differences in gender, smoking status, and stage among different subtypes. And there was no statistically significant difference in histological diagnosis and age among subtypes (Table S3).

**Fig. 3:**
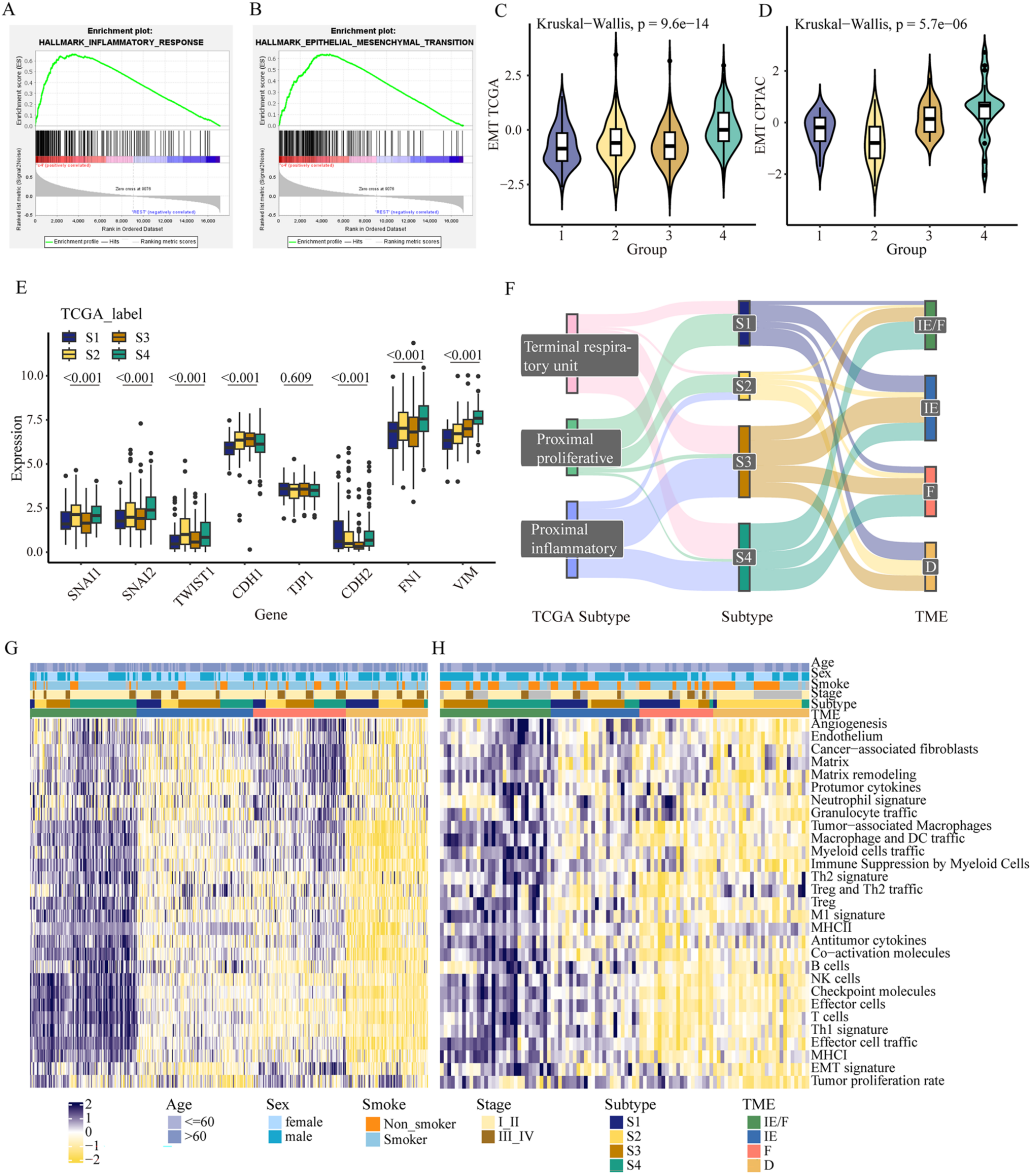
Biological characteristics and tumor microenvironment (TME) of DMGs-associated subtypes. **A**–**B** GSEA analysis indicated that S4 exhibited significant inflammatory response and epithelial mesenchymal transition enrichment. **C**–**D** The difference of EMT scores in TCGA and CPTAC. **E** The expression of EMT-associated genes in subgroups, including CDH1, CDH2, FN1, SNAI1, SNAI2, TJP1, TWIST1 and VIM. **F** Sankey relational diagram for the changes of molecular subtypes, DMGs-associated multi-omics subtypes, TME subtypes and clinical features. **G**–**H** The heatmap of the abundance of 29 immune cell infiltration signature in TCGA and CPTAC. Age, sex, smoke, stage, subtype and TME subtype are shown as patient annotations, and gray indicates missing value

In our study, 15 differential DMGs among multi-omics subtypes were identified in TCGA. The differential multi-omics expression of DMGs were shown in Fig. 2G–H. It was evident that high methylation expression of DMGs corresponded to low mRNA expression. SYCP2L, FOXI3 and CALCA exhibited high expression of mRNA and low methylation values in S1. Genes such as ZSCAN1 and ZNF135 showed high expression of mRNA and low expression of methylation in S2, respectively. The most of DMGs have low mRNA and high methylation expression in S3, and GATA3 expressed high mRNA and low methylation expression in S4. In addition, the expression patterns of subtypes in the CPTAC cohort were similar to TCGA cohort (Fig. 2I–J), providing further validation of the reliability of the subtypes.

### The molecular characterization and tumor microenvironment of multi-omics subtype

We evaluated the biological functions of DMGs-associated multi-omics subtype with Gene set enrichment analysis (GSEA) and results were shown in Table S4. The GSEA results showed enrichment of pathways such as G2M checkpoint, E2F targets, and MYC target V1 in subtype S2. S4 was markedly enriched in both immune regulation and stromal-related signaling pathways such as IL-6/JAK/STAT3 pathway, interferon γ response, epithelial mesenchymal transition signaling (Fig. 3A–B). Meanwhile, the activation of EMT in S4 was also validated by the results of EMT score and EMT related genes expression in TCGA and CPTAC (Fig. 3C–E).

In this study, tumor-infiltrating of immune cells were evaluated by ssGSEA, and consensus clustering was performed to find TME clustering. There were significant differences in the TME of four DMGs multi-omics subtypes in TCGA (Fig. 3G). S3 was found to be concentrated mainly in immune-enriched, non-fibrotic (IE) TME subtype, exhibiting a more favorable TME with immune infiltration. S4 was found to be concentrated mainly in immune-enriched, fibrotic (IE/F) TME subtype with high immune and stromal infiltration. Similarly, the TME features in DMGs multi-omics subgroups were validated by the CPTAC dataset (Fig. 3H).

The association between DMGs multi-omics subgroups and existing molecular classifications and features were depicted in Fig. 3F. The S3 subtypes had a higher proportion of proximal inflammatory (PI) samples, while the S4 subtype predominantly consisted of terminal respiratory unit (TRU) samples and PI samples. In the S1 and S2 subtypes, there was a higher proportion of proximal proliferative (PP) samples compared to other subtypes. We also observed distinct TME features across the DMGs multi-omics subgroups. The S1 subtype may exhibit characteristics of both the IE and D subtypes. The S2 subtype tended to concentrate within D subtype, while S3 subtype was characterized by features resembling the IE subtype. And S4 subtype did not demonstrate characteristics associated with D subtype.

### Immune characteristics and therapy efficiency in subtypes

**Fig. 4:**
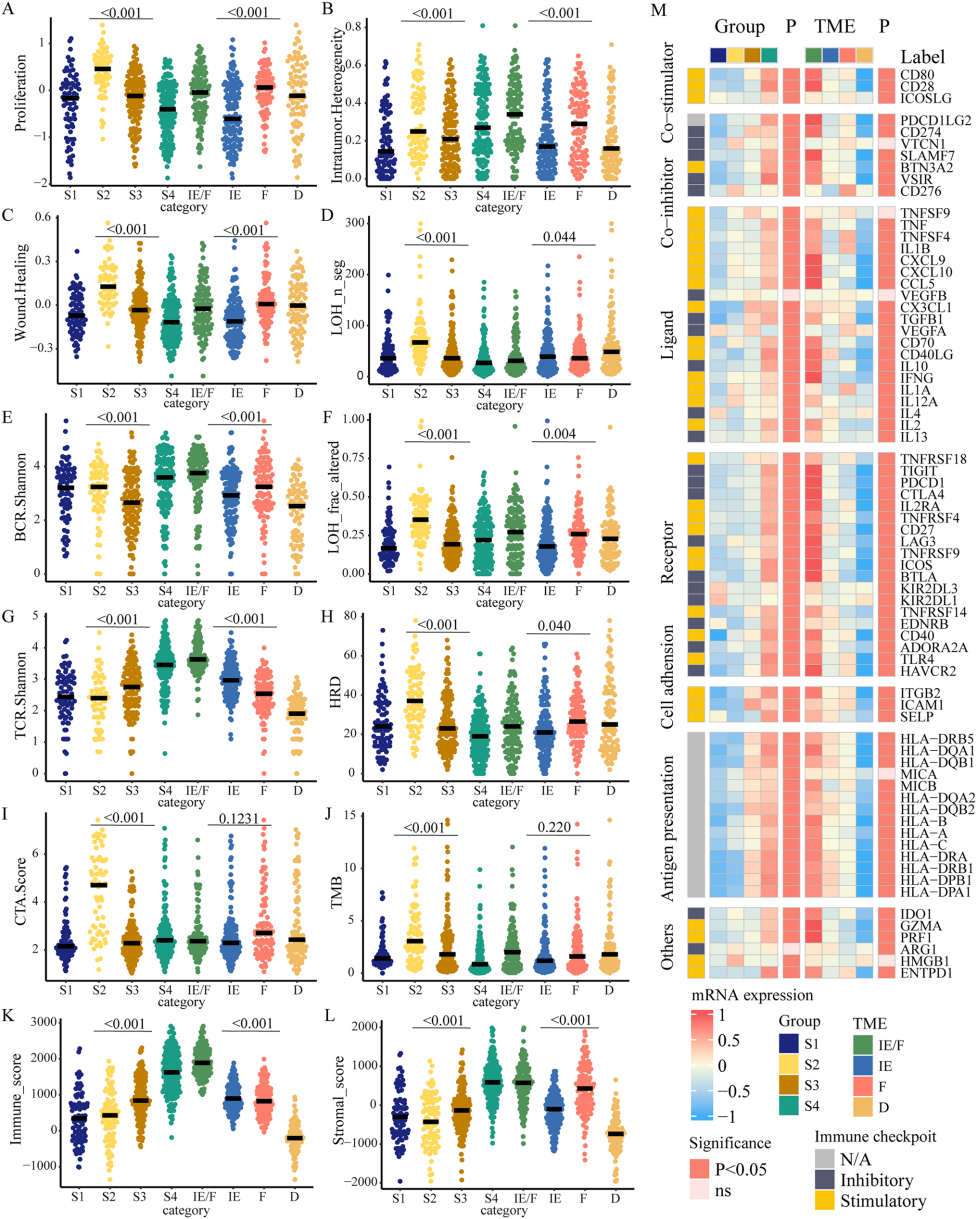
The immune characteristics of DMGs-associated multi-omics subtypes and TME subtypes in TCGA. **A**–**L** The difference among subtypes in proliferation score (**A**), ITH score (**B**), wound healing score (**C**), LOH n seg (**D**), BCR Shannon index (**E**), LOH trac altered (**F**), TCR Shannon index (**G**), homologous recombination deficiency (**H**), CTA score (**I**), TMB (**J**), immune score (**K**) and stromal score (**L**). **M** Heatmap depicting the mean values of mRNA expressions of immune modulators genes among distinct DMGs-associated subgroups and TME subgroups. The statistical difference among subtypes is compared by Kruskal–Wallis test. A two-sided p < 0.05 was regarded statistically significant

Potential factors determining tumor immunogenicity were analyzed in our study. S1 had the lowest intratumor heterogeneity (ITH). High proliferation, wound healing, CTA score and HRD were shown in S2. S4 exhibited the highest BCR/TCR diversity (BCR/TCR Shannon) and S3 showed relatively high TCR diversity (Fig. 4A–J). Additionally, the immune score and stromal score were compared among subtypes. S3 and S4 displayed elevated stromal and immune scores, whereas S1 and S2 showed comparatively lower stromal and immune scores (Fig. 4K–L).

**Fig. 5:**
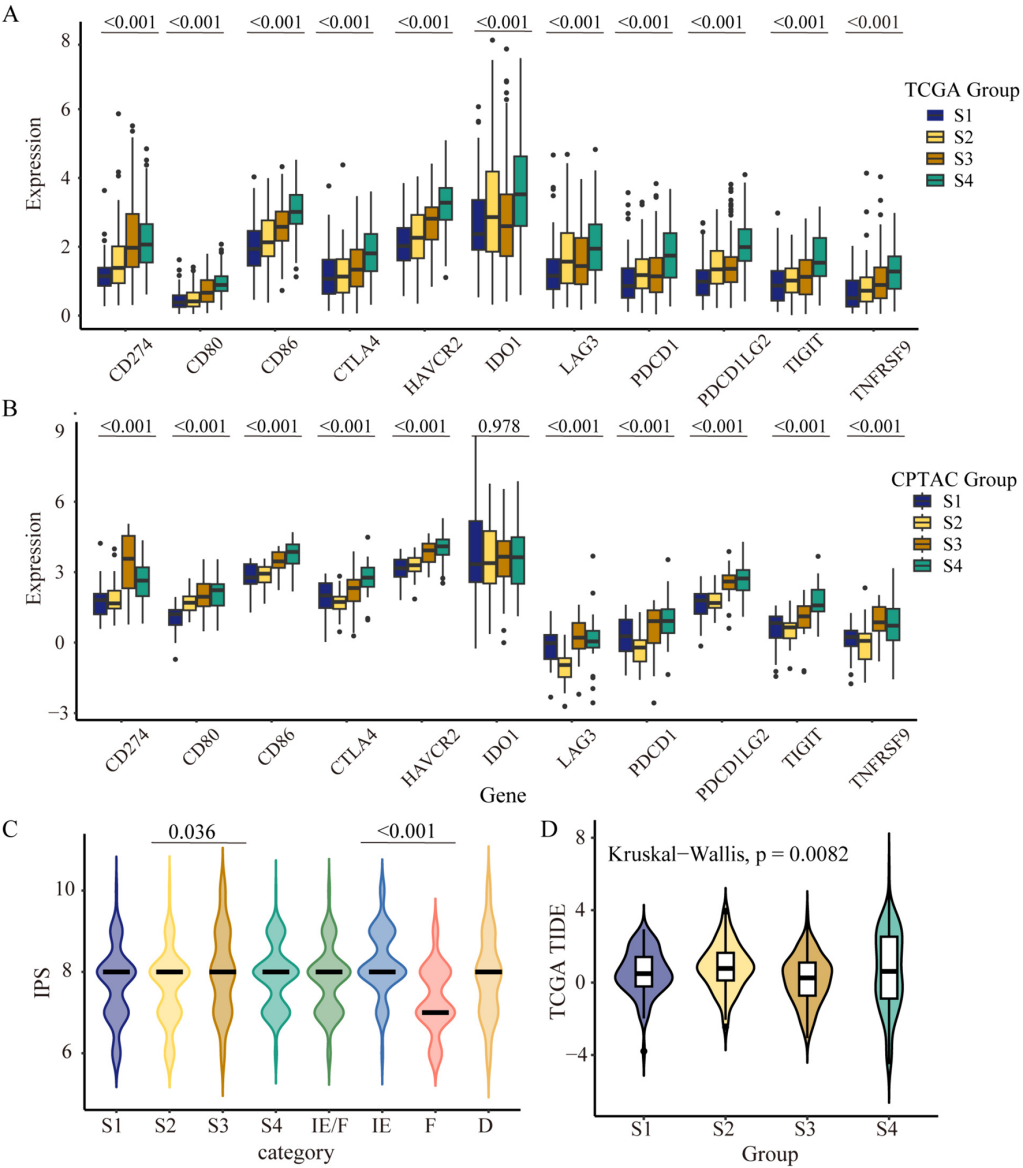
The expression of immunotherapy predictors in different DMGs-associated multi-omics subtypes. **A**–**B** The expression of immune checkpoint genes among subgroups in TCGA and CPTAC, respectively. **C** The immunophenoscore (IPS) in different subgroups and TME subtypes. **D** Differential expression of Tumor Immune Dysfunction and Exclusion (TIDE) score among subtypes. The statistical difference among subtypes is compared by Kruskal–Wallis test. A two-sided *P* < 0.05 was regarded statistically significant

The comparison of the expression of immune modulators was shown in Fig. 4M. The differences in immune modulators expression among subtypes exhibited statistical significance excepted VEGFB and ARG1. Most immune modulators exhibited upregulation in S3 and S4 and downregulation in S1 and S2. Additionally, we compared the immune modulators expression among the four TME subtypes. We found that immune modulators were upregulated in IE and IE/F, while downregulated in subtype D. Further comparison of DMGs-associated multi-omics subtypes showed that the immune checkpoints were high expression in S3 and S4, and low expression in S1 and S2 in TCGA and CPTAC (Fig. 5A–B). The comparison among subgroups by IPS and TIDE showed that significant differences were observed among the subgroups. IPS was elevated in S3 and TIDE had a lower tendency in the S3 (*P* = 0.036, *P* = 0.0082; Fig. 5C–D).

Additionally, the mutation and co-occurring mutation results of targeted therapy genes were shown in Table 1. Most of the mutation and co-occurring alterations were statistically significant among four subgroups, except for co-occurring mutation of EGFR & TP53 and STK11 & TP53. The highest and lowest mutation frequency of EGFR was observed in S4 and S1, respectively. The mutation frequencies of STK11 and KRAS genes were significantly higher in S1. The mutation frequency of TP53 was significantly higher in S2. The highest co-occurring mutation of KRAS & TP53 and the lowest co-occurring mutation of KRAS & STK11 were found in S3. There was a tendency towards a higher co-occurring mutation frequency of EGFR & TP53 in S2.

**Table 1:** The differences in mutation frequencies of targeted therapy genes among DMGs-associated multi-omics subtypes in TCGA

Variables	S1	S2	S3	S4	*P*
n	83	80	126	146	
EGFR (%)	1 (1.2)	10 (12.5)	13 (10.3)	25 (17.1)	0.003
STK11 (%)	44 (53.0)	9 (11.2)	2 (1.6)	14 (9.6)	< 0.001
TP53 (%)	17 (20.5)	59 (73.8)	74 (58.7)	64 (43.8)	< 0.001
KRAS (%)	44 (53.0)	9 (11.2)	41 (32.5)	28 (19.2)	< 0.001
EGFR & TP53 (%)	1 (1.2)	9 (11.2)	7 (5.6)	10 (6.8)	0.064
KRAS & TP53 (%)	7 (8.4)	4 (5.0)	26 (20.6)	11 (7.5)	0.001
KRAS & STK11 (%)	27 (32.5)	2 (2.5)	0 (0.0)	4 (2.7)	< 0.001
STK11 & TP53 (%)	5 (6.0)	5 (6.2)	1 (0.8)	9 (6.2)	0.119

## Discussion

This study identified 147 driver methylation genes (DMGs) and obtained a novel multi-omics integrated DMG-associated subtype with SNF in LUAD. Overall, the S2 subtype showed poorer prognosis. S3 and S4 subtypes exhibited increased immune infiltration and high expression of immune checkpoint genes. Simultaneously, S4 showed characteristics of stromal infiltration. Therefore, S3 could be a potential beneficial subgroup for immunotherapy.

Up to date, numerous studies mainly focused on exploring the molecular subtypes of LUAD. The TCGA project proposed mRNA-based subtypes of LUAD, including proximal inflammatory (PI), proximal proliferative (PP) and terminal respiratory unit (TRU), which provided a foundation for further research into its molecular pathogenesis (Network 2014). Gillette et al. integrated proteogenomics to expose multi-omics clusters and immune subtypes of LUAD (Gillette et al. 2020). Xu et al. proposed a methylation-based subtypes that could predict the prognosis and provide potential drug targets to develop effective immunotherapies for LUAD (Xu et al. 2020). These studies have not taken into account the molecular subtypes and characteristics of LUAD from the perspective of driver methylation and multi-omics simultaneously. Our research identified and validated DMGs in LUAD. Based on multi-omics data of DMGs, a novel LUAD molecular subtype was determined to predict prognosis and treatment response.

Given the aggressive time line and resistance to standard chemotherapy of LUAD, a primary objective had been to discover additional pharmacological targets and enrichment populations that provide insights into personalized treatment and improve the prognosis of LUAD patients. The identified DMGs could clearly distinguish LUAD from normal tissue, and the negative correlation was consistent with the silencing of methylation-induced of key regulatory genes (Pfeifer 2018). In our study, GATA3 exhibited high mRNA expression in S2 and S4 subtypes, which was associated an unfavorable prognosis in LUAD (Hashiguchi et al. 2017). The expression of IGF2BP1, an oncogene and potential therapeutic target for cancers (Huang et al. 2018; Wu et al. 2022), differed among the four subtypes. We found a higher silhouette coefficient in multi-omics than single-omics analysis, suggesting that superior performance compared to single-omics analysis.

The present study showed that S2 had the poorest survival prognosis, highlighting the importance of increased clinical surveillance and the implementation of corresponding measures to prevent disease recurrence and progression in S2 subtype. Our study found that MYC targets V1 and V2, DNA repair, and G2M checkpoint pathways were enriched in S2, indicting a potential association with poor prognosis (Schulze et al. 2020; Oshi et al. 2021, 2020). S4 exhibited enrichment in immune regulation and stromal-related signaling pathways, such as inflammatory response, INF-γ, and epithelial mesenchymal transition pathways. Intriguingly, the validation of EMT activation in S4 was supported by the findings of EMT score and EMT-associated genes, suggesting its potential association with tumor progression and metastasis (Smith and Bhowmick 2016).

We observed higher expression of immune checkpoints in S3 and S4 subtypes. While only S3 showed higher IPS and lower TIDE scores, suggesting S3 rather than S4 had potential sensitivity to immune checkpoint inhibitors (ICIs). The reason may be that S3 subtype was predominantly enriched in IE with high immune infiltration, and the S4 patients were primarily distributed in the IE and IE/F subtypes with high immune and stromal infiltration, suggesting a different tumor microenvironment (TME) between S3 and S4. The growing evidence revealed that genomic alterations in oncogenic drivers and tumor suppressor genes play a crucial role in the molecular diversity of NSCLC. The alterations could help predict and prevent the emergence of acquired resistance, and facilitate the development of novel personalized treatments (Skoulidis and Heymach 2019). We analyzed the mutation and co-occurring mutation of oncogenic drivers and tumor suppressor genes, including EGFR, KRAS, TP53, and SKT11. The high co-occurring mutation frequency of KRAS & TP53 and the low co-occurring mutation frequency of KRAS & STK11 were found in S3, suggesting that it may be a beneficial group for immunotherapy (Skoulidis and Heymach 2019; Gu et al. 2021; Shen et al. 2022).

Our study had several strengths. First, we identified and validated DMGs in different database of LUAD, which provided some potential therapy targets. Second, we developed a novel molecular subtype that facilitated the identification of treatment-sensitive populations by integrating multi-omics of DMGs. Third, the molecular characteristics of subtypes were validated from multiple perspectives, which provided relatively complete evidence. Meanwhile, some potential limitations still needed to be acknowledged in our study. First, our study was based on public datasets and it could need a further validation by population-based data. Second, the identification of DMGs and novel subtypes were primarily relied on bioinformatics analysis, the results required additional in vitro and in vivo validation. Third, the clinical features such as radiological features of LUAD need further analysis and comparison among subgroups. Forth, in addition to immunotherapy predictive biomarkers, the response to immunotherapy still requires validation through experiments and clinical trials. Fifth, bulk data might ignore variations among different cell types (Shapiro et al. 2013), the findings could be analyzed and validated with single-cell sequencing. Sixth, further validation is needed to promote translation. The focus of our future research will be on the collection of multi-omics data from LUAD cohorts and the development of R packages.

## Conclusion

Our research identified and validated driver methylation genes (DMGs) of LUAD. And we comprehensively profiled the role of distinct DMGs-associated multi-omics subtypes in clinical implication and molecular features in LUAD. The DMGs- associated subtypes of LUAD demonstrate prognostic predictive ability that could help inform treatment response and provide complementary information to the existing subtypes.

### Supplementary Information

Below is the link to the electronic supplementary material.

Supplementary file1 (TIF 9016 KB)

Supplementary file2 (XLSX 30 KB)

## Data Availability

Publicly available datasets were analyzed in this study. These data can be downloaded from TCGA database (https://xenabrowser.net/datapages/) and the supplementary materials from article “Proteogenomic characterization reveals therapeutic vulnerabilities in lung adenocarcinoma” (https://www.ncbi.nlm.nih.gov/pmc/articles/PMC7373300/).
