# Supplementary figures and images for: A novel subtype based on driver methylation–transcription in lung adenocarcinoma
Source: J Cancer Res Clin Oncol. 2024 May 22;150(5):269. doi: 10.1007/s00432-024-05786-3 (PMC11111506; doi:10.1007/s00432-024-05786-3)

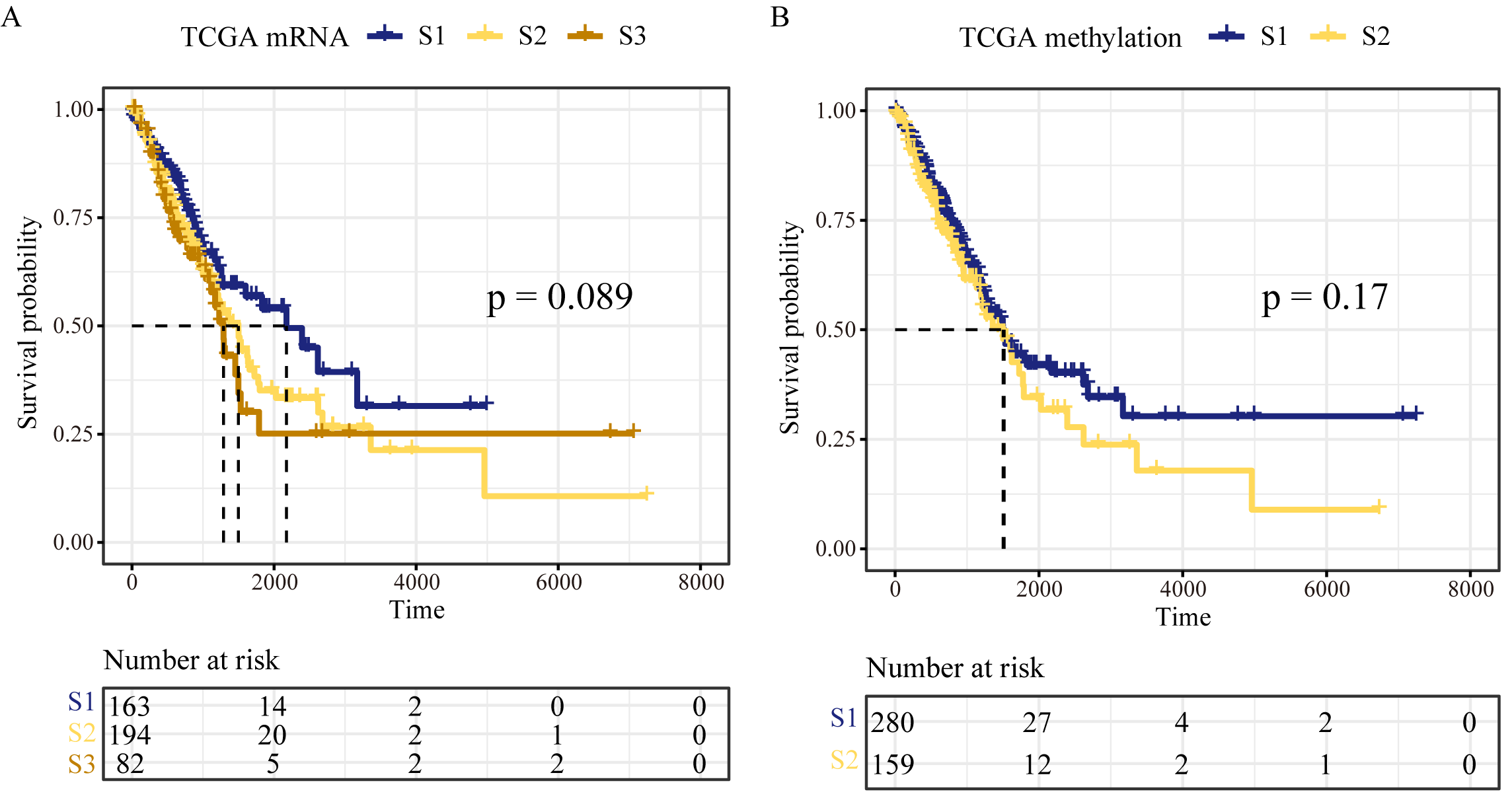

Supplement: Supplementary file 1 — Supplementary file1 (TIF 9016 KB) [file 432_2024_5786_MOESM1_ESM.tif]
